# Chondrosarcoma patient characteristics, management, and outcomes based on over 5,000 cases from the National Cancer Database (NCDB)

**DOI:** 10.1371/journal.pone.0268215

**Published:** 2022-07-28

**Authors:** Taylor D. Ottesen, Blake N. Shultz, Alana M. Munger, Michael Amick, Courtney S. Toombs, Gary E. Friedaender, Jonathan N. Grauer

**Affiliations:** 1 Department of Orthopaedics and Rehabilitation, Yale School of Medicine, New Haven, CT, United States of America; 2 Harvard Combined Orthopaedics Residency Program, Boston, MA, United States of America; Mie University Graduate School of Medicine, JAPAN

## Abstract

**Introduction:**

Chondrosarcoma, although relatively uncommon, represents a significant percentage of primary osseous tumors. Nonetheless, there are few large-cohort, longitudinal studies of long-term survival and treatment outcomes of chondrosarcoma patients and none using the National Cancer Database (NCDB).

**Methods:**

Chondrosarcoma patients were identified from the 2004–2015 NCDB datasets and divided on three primary tumor sites: appendicular, axial, and other. Demographic, treatment, and long-term survival data were determined for each group. Multivariate Cox analysis and Kaplan-Meier survival curves were generated to assess long-term survival over time for each.

**Results:**

In total, 5,329 chondrosarcoma patients were identified, of which 2,686 were appendicular and 1,616 were axial. Survival was higher among the appendicular cohort than axial at 1-year, 5-year, and 10-year (89.52%, 75.76%, and 65.24%, respectively). Multivariate Cox analysis identified patients in the appendicular cohort to have significantly greater likelihood of death with increasing age category, distant metastases at presentation, and male sex (p<0.001 for each). Best outcomes for seen for those undergoing surgical treatment (p<0.001). Patients in the axial cohort were with increased likelihood of death with increasing age category and distant metastases (p<0.001), while surgical treatment with or without radiation were associated with a significant decrease (p<0.001). Kaplan-Meier survival analysis showed worst survival for the axial cohort (p<0.001) and patients with distant metastases at presentation (p<0.001). Survival was not significantly different between older (2004–2007) and more recent years (2012–2016) (p = 0.742).

**Conclusions:**

For both appendicular and axial chondrosarcomas, surgical treatment remains the mainstay of treatment due to its continued superiority for the long-term survival of patients, although advancements in survival over the last decade have been insignificant. Presence of distant metastases and axial involvement are significant, poor prognostic factors perhaps because of difficulty in surgical excision or extent of disease.

## Introduction

Chondrosarcomas represent a heterogenous group of malignant bone tumors characterized by the formation of hyaline cartilaginous neoplastic tissue [1]. Following osteosarcoma, chondrosarcoma is the second-most common primary solid tumor of bone with 3 new cases per 10^6^ population per year [2]. Chondrosarcomas primarily affect adults, with the incidence rising with increasing patient age [3]. The most common anatomical location of origin is pelvis, followed by the proximal femur, proximal humerus, distal femur and ribs [4]. The clinical presentation for patients with chondrosarcoma include pain (especially at night), pathologic fracture, or an incidental finding [5], with the lungs representing the most common site of metastasis. In general, up to 26% of patients developed distant metastasis [6, 7].

The survival rate of patients with chondrosarcoma is higher than that of osteosarcoma and Ewing sarcoma patients, with 5-year survival rates between 72–75% and 10-year survival rates of 69% [6, 8, 9]. The gold standard treatment for chondrosarcomas has become wide surgical excision–which has largely remained unchanged in recent decades. However, for Grade I chondrosarcomas, prior studies have shown no difference in overall survival of patients treated with wide excision versus those treated with intra-lesional curettage [10]. For more aggressive subtypes, surgical resection with wide margins has been associated with a longer duration of survival in comparison to marginal or intralesional resections [11]. This may be because chondrosarcomas are resistant to both chemotherapy and radiotherapy due to the extracellular matrix, low percentage of dividing cells, and poor vascularity [12].

Outcomes for patients with chondrosarcoma depend on grade, stage, and surgical margins with higher grade showing decreased patient survival [2, 13]. Despite advances and many different efforts to try to refine treatment options, the survival rates have largely remained the same in recent decades. Prior studies have been single institution studies or used the SEER (Surveillance, Epidemiology and End Results) database and mainly investigated median survival and variables associated with poor prognosis [14, 15]. To-date there is only one large study of chondrosarcoma analyzing 2,890 cases of chondrosarcoma from the SEER database [16], however, the study looked at cases originating 50 years ago from 1973 to 2003. At this time, no study has analyzed chondrosarcomas within The National Cancer Database (NCDB).

NCDB, created by the American College of Surgeons and American Cancer Society, is a multi-institutional dataset of over 30 million patient variables containing the demographic and outcome variables of over 70% of new cancer diagnoses in the United States [17, 18]. With the advent of many new medical and surgical oncologic treatments, the current study seeks to use newer data from the large, robust NCDB database to: (1) refresh the current literature, (2) understand the recent incidence of chondrosarcoma and (3) evaluate the demographic, lesion, and treatment variables associated with improved outcomes in patients with chondrosarcoma.

## Materials and methods

### Data source and study population

The NCDB was utilized to conduct a retrospective database cohort study of all patients diagnosed with chondrosarcoma from 2004–2015. A joint initiative by the American College of surgeons and the American Cancer Society, the NCDB tracks treatments and outcomes of patients with malignant neoplastic diseases to improve the quality of care for cancer patients. The database contains hospital registry data from more than 1,500 Commission on Cancer (CoC)-accredited facilities, representing more than 70% of newly diagnosed cancer cases nationwide [17, 19].

Chondrosarcoma diagnosis was based on the International Classification of Diseases for Oncology, 3^rd^ edition (ICD-O-3) using the histology codes 9220, 9221, 9243, 9240, and 9242. Patients were then separated into three different groups based on location of primary tumor: appendicular (C40.0, C40.1, C40.2, C40.3, and C40.9), axial including pelvis (ICD-O-3 codes C41.0, C41.1, C41.2, and C41.4), and other location (C40.8, C41.3, C41.8, and C41.9) (e.g. head, neck, or mandible). In order to be compliant with NCDB use guidelines, we combined anatomical sites into these 3 categories to allow for robust analysis, and the avoidance of small samples which is a required protocol when working with NCDB data.

Patients were excluded from analysis if treatment location of the patient was different that the reporting facility (Class of Case = 00), or if data about presence of metastasis at time of diagnosis was missing.

### Population characteristics

Demographics including age, sex, Charles-Deyo score, and metastasis at presentation were identified from the database and divided into three groups based on the year of diagnosis: 2004–2007, 2008–2011, and 2012–2015.

Treatment data for surgery, radiation, chemotherapy, and combination treatments were also extracted from the database. Finally, survival for each primary site cohort was calculated for one-, five-, and ten-years.

### Comparison of populations

Comparative analyses of preoperative and treatment variables were performed using bivariate analyses between the axial, appendicular, and other cohorts. Categorical variables were tested using Pearson’s chi-squared test, continuous variables using one-way ANOVA, and ordinal variables (ASA class) using Kruskal-Wallis test.

Multivariate Cox analysis was performed on the axial and appendicular cohort to determine the influence of preoperative variables and treatment choice on the likelihood of death. Incidence rate ratio (IRR) was then used to determine the relative risk for the population over time, taking into account the shifting population as a result of deaths or remission [20]. IRR was calculated by dividing the incidence rate defined as the number of events person-years of the exposed population by the total of all people at risk in this case for death at any one point in time. This aimed to more accurately determine the relative effect of exposures on the risk of occurrence among the exposed and unexposed populations.

Lastly, the long-term survival among cohorts was analyzed used Kaplan Meier survival curves. Survival curves were presented to compare survival among patients with and without distant metastases at presentation, patients across the appendicular, axial, or other cohorts, and patients diagnosed in 2004–2007, 2008–2011, and 2012–2015.

All analyses were performed using Stata® version 13.0 (StataCorp, LP, College Station, Texas, USA). Significance was set at p<0.05. This study was given exemption by our institution’s Human Investigations Committee.

## Results

### Demographics

In total, 5,329 chondrosarcoma patients were identified, of which appendicular represented 2,686, axial represented 1,616, and other represented 1,027. Mean age ± standard deviation for the total cohort was 52.11 ± 17.51 and 47.59% of the patients were female (Table 1).

**Table 1:** Comparison of demographics between the appendicular, axial, and other osteosarcoma cohorts.

Patient Characteristic	Total	Appendicular	Axial	Other	p-value
Number of patients	5,329	2,686	1,616	1,027	
**Mean age in years (± SD)**	52.11 ± 17.51	52.57 ± 17.49	50.35 ± 17.60	53.66 ± 17.21	**< 0.001**
**Females (%)**	2,536 (47.59)	1,399 (52.08)	720 (44.55)	417 (40.60)	**< 0.001**
**Charles-Deyo Score (%)**					**0.023**
0	4,436 (83.24)	2,228 (82.95)	1,383 (85.58)	825 (80.33)	
1	699 (13.12)	366 (13.63)	175 (10.83)	158 (15.38)	
2	154 (2.89)	73 (2.72)	46 (2.85)	35 (3.41)	
3	40 (0.75)	19 (0.71)	12 (0.74)	9 (0.88)	
**Metastasis at presentation (%)**					0.531
No	4,998 (93.79)	2,527 (94.08)	1,515 (93.75)	956 (93.09)	
Yes	331 (6.21)	159 (5.92)	101 (6.25)	71 (6.91)	
Year of diagnosis* (%)					0.807
2004–2007	1,803 (33.83)	921 (34.29)	549 (33.97)	333 (32.42)	
2008–2011	1,921 (36.05)	961 (35.78)	575 (35.58)	385 (37.49)	
2012–2015	1,605 (30.12)	804 (29.93)	492 (30.45)	309 (30.09)	

SD = standard deviation

Differences in age between the groups was observed (p<0.001), with the average age of the axial cohort being slightly younger and the “other” category patients being slightly older (Table 1). There was difference in sex percentage between groups (p<0.001) with more female patients in the axial cohort than the appendicular or other groups. Charles Deyo Score was reported as zero for 83.24% of overall study population; the axial cohort had the largest percentage of scores of zero compared to the appendicular and other groups.

Metastatic disease was present at diagnosis for 6.21% of all patients (and not different for the different tumor sites). The cohort was distributed evenly across time periods, with approximately 1/3 of the cohort arising from each time period (again, not different for the different tumor sites). The mean “last contact or death” time from diagnosis was 55.36 months with a standard deviation of 38.51 months. The median “last contact or death” time from diagnosis was 48.62 months.

### Treatment

Surgical treatment was different between the three groups. While the vast majority of patients in all three groups underwent surgery, there was a significantly difference between the groups (p<0.001) with more patients in the appendicular group having surgery (93.04%) than in the axial group (85.33%) (p<0.001, Table 2).

**Table 2:** Comparison of operative data and long-term survival for the appendicular and axial cohorts.

Patient Characteristic	Total	Appendicular	Axial	Other	p-value
Number of patients	5,329	2,686	1,616	1,027	
**Surgical treatment**					**< 0.001**
No Surgery	511 (9.59)	186 (6.92)	235 (14.54)	90 (8.76)	
Surgery	4,814 (90.34)	2,499 (93.04)	1,379 (85.33)	936 (91.14)	
Unknown	4 (0.08)	1 (0.04)	2 (0.12)	1 (0.10)	
**Radiation Therapy**					**< 0.001**
None	4,747 (89.08)	2,534 (94.34)	1,312 (81.19)	901 (87.73)	
Preoperative	38 (0.71)	13 (0.48)	17 (1.05)	8 (0.78)	
Postoperative	439 (8.24)	78 (2.90)	264 (16.34)	97 (9.44)	
Both or Intraoperative	4 (0.08)	1 (0.04)	2 (0.12)	1 (0.10)	
Unknown	101 (1.90)	60 (2.23)	21 (1.30)	20 (1.95)	
**Chemotherapy**					**0.001**
No (0)	4,737 (88.89)	2,373 (88.35)	1,441 (89.17)	923 (89.87)	
Yes (1)	368 (6.91)	183 (6.81)	130 (8.04)	55 (5.36)	
Unknown (2)	224 (4.20)	130 (4.84)	45 (2.78)	49 (4.77)	
**Treatment Combination**					
No treatment	634 (11.90)	290 (10.80)	229 (14.17)	115 (11.20)	**< 0.001**
Isolated surgery	3,925 (73.65)	2,153 (80.16)	1,003 (62.07)	769 (74.88)	
Isolated radiation	9 (0.17)	2 (0.07)	3 (0.19)	4 (0.39)	
Isolated chemotherapy	93 (1.75)	28 (1.04)	48 (2.97)	17 (1.66)	
Surgery and radiation	398 (7.47)	62 (2.31)	251 (15.53)	85 (8.28)	
Surgery and chemotherapy	202 (3.79)	124 (4.62)	57 (3.53)	21 (2.04)	
Chemotherapy and radiation	1 (0.02)	1 (0.04)	0 (0.00)	0 (0.00)	
Surgery, radiation, and chemotherapy	67 (1.26)	26 (0.97)	25 (1.55)	16 (1.56)	
**Survival**					**< 0.001**
1-yr survival (%)	90.38	89.52	89.66	93.75	
5-yr survival (%)	75.61	75.76	72.14	80.59	
10-yr survival (%)	64.18	65.24	60.12	67.67	

Radiation therapy was much less commonly used across all groups. In the appendicular group, less than 1% underwent pre-operative radiation and 2.90% post-operative, compared to the axial group where 1.05% also underwent pre-operative but 16.34% underwent post-operative radiation (p<0.001, Table 2).

Chemotherapy was not commonly used as well, with 5–8% of each group using this modality. There was a significant difference between groups for the receipt of chemo with the axial group having the highest at 8.04%, appendicular at 6.81%, and other at 5.36% (p<0.001, Table 2). Chemotherapy was not significantly associated with a favorable prognosis (p = 0.076 for the equality of survivor functions).

Most patients in the study had only surgical treatment performed (73.7%). For the appendicular cohort, 80.16% of patients had only surgery, 4.62% of patients had surgery and chemotherapy and 2.31% had surgery and radiation. This was in contrast to the axial group where 62.07% of patients had surgery only, 15.53% had surgery and radiation and 3.53% had surgery and chemotherapy. (p<0.001, Table 2). The other group had rates of 74.88% surgery only, 8.28% surgery and radiation and 2.04% surgery and chemotherapy (p<0.001, Table 2).

### Survival analyses

Survival for the overall cohort was 90.38% at 1 year, 75.61% at 5 years and 64.18% at 10 years. Survival rates were highest in the “other” cohort (93.75% at 1 year, 80.59% at 5, 67.67% at 10) compared to the appendicular and axial cohorts (p<0.001, Table 2).

The multivariate Cox analysis for the appendicular cohort revealed the likelihood of death to be significantly increased with age category and distant metastases at presentation (IRR = 6.794, p<0.001) and significantly decreased for female sex (IRR = 0.692, p<0.001), and surgical treatment (IRR = 0.533, p<0.001, Table 3). Year of diagnosis and treatment choice were not significantly related to likelihood of death.

**Table 3:** Multivariate Cox analysis of the likelihood of death at any given time for demographic and operative variables for the appendicular cohort.

Likelihood of death at any given time	IRR	95% Confidence Interval			p-value
**Age Category**					
< 23	Ref	Ref		Ref	Ref
23–45	2.046	0.940	-	4.455	**0.071**
46–62	3.747	1.751	-	8.019	**0.001**
62+	9.295	4.286	-	20.158	**< 0.001**
**Sex**					
Male	Ref	Ref		Ref	Ref
Female	0.692	0.592	-	0.810	**< 0.001**
**Metastasis at time of diagnosis**					
Yes	6.794	5.389	-	8.565	**< 0.001**
**Year of diagnosis**					
2004–2007	Ref	Ref		Ref	Ref
2008–2011	1.081	0.899	-	1.300	0.406
2012–2015	0.965	0.777	-	1.200	0.750
**Treatment choice**			-		
None	Ref	Ref		Ref	Ref
Surgery	0.533	0.414	-	0.685	**< 0.001**
Radiation	3.733	0.507	-	27.455	0.196
Chemotherapy	2.165	1.326	-	3.536	**0.002**
Surgery and radiation	1.234	0.836	-	1.820	0.290
Surgery and chemotherapy	1.042	0.748	-	1.451	0.807
Radiation and chemotherapy	7.791	1.056	-	57.496	**0.044**

IRR = incidence rate ratio

For the axial cohort, similar analysis revealed the likelihood of death to be significantly increased with increasing age category and distant metastases (Incident Rate Ratio (IRR) = 3.12, p<0.001), while it was significantly decreased with surgical treatment (IRR = 0.356, p<0.001) and surgical treatment with radiation (IRR = 0.373, p<0.001) (Table 4). There were not enough patients who received radiation only or radiation plus chemotherapy for those variables to be analyzed. There was also a small and statistically insignificant improvement in likelihood of death in those patients treated in the later years of the data collection.

**Table 4:** Multivariate Cox analysis of the likelihood of death at any given time for demographic and operative variables for the axial cohort.

Likelihood of death at any given time	IRR	95% Confidence Interval			p-value
**Age Category**					
< 23	Ref	Ref		Ref	Ref
23–45	2.153	1.171	-	3.9555	**0.014**
46–62	3.635	2.013	-	6.562	**< 0.001**
62+	5.829	3.113	-	10.914	**< 0.001**
**Sex**					
Male	Ref	Ref		Ref	Ref
Female	0.892	0.736	-	1.081	0.244
**Metastasis at time of diagnosis**					
Yes	3.122	2.327	-	4.189	**< 0.001**
**Year of diagnosis**					
2004–2007	Ref	Ref		Ref	Ref
2008–2011	0.926	0.748	-	1.146	0.480
2012–2015	0.680	0.513	-	0.900	**0.007**
**Treatment choice**					
None	Ref	Ref		Ref	Ref
Surgery	0.356	0.278	-	0.458	**< 0.001**
Radiation	-	-	-	-	**-**
Chemotherapy	1.971	1.299	-	2.991	**0.001**
Surgery and radiation	0.373	0.265	-	0.523	**< 0.001**
Surgery and chemotherapy	1.694	1.143	-	2.510	**0.009**
Radiation and chemotherapy	-	-	-	-	-

IRR = incidence rate ratio

**Fig 1:**
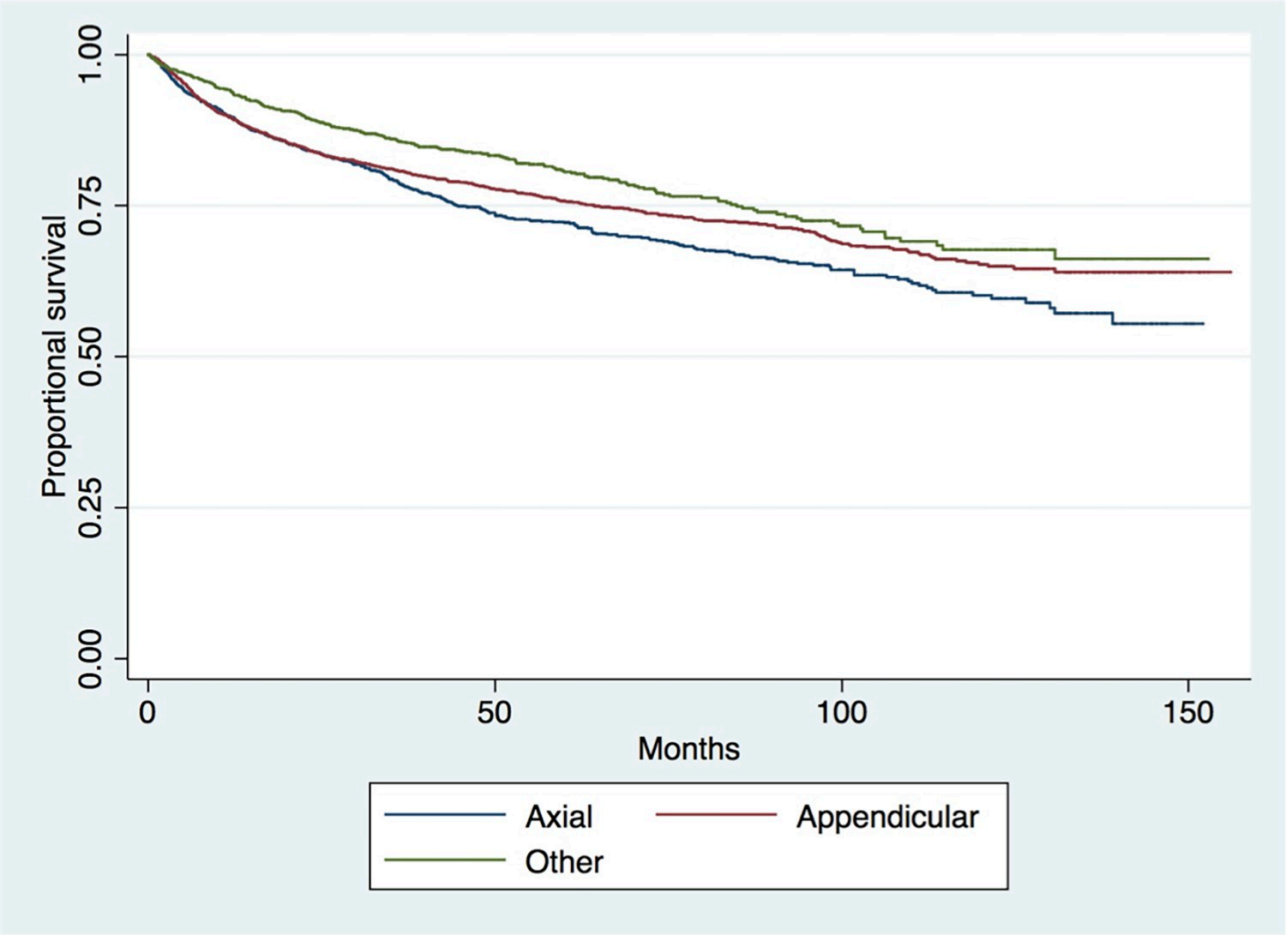
Long-term survival of patients in the axial, appendicular, and other cohorts (p<0.001).

**Fig 2:**
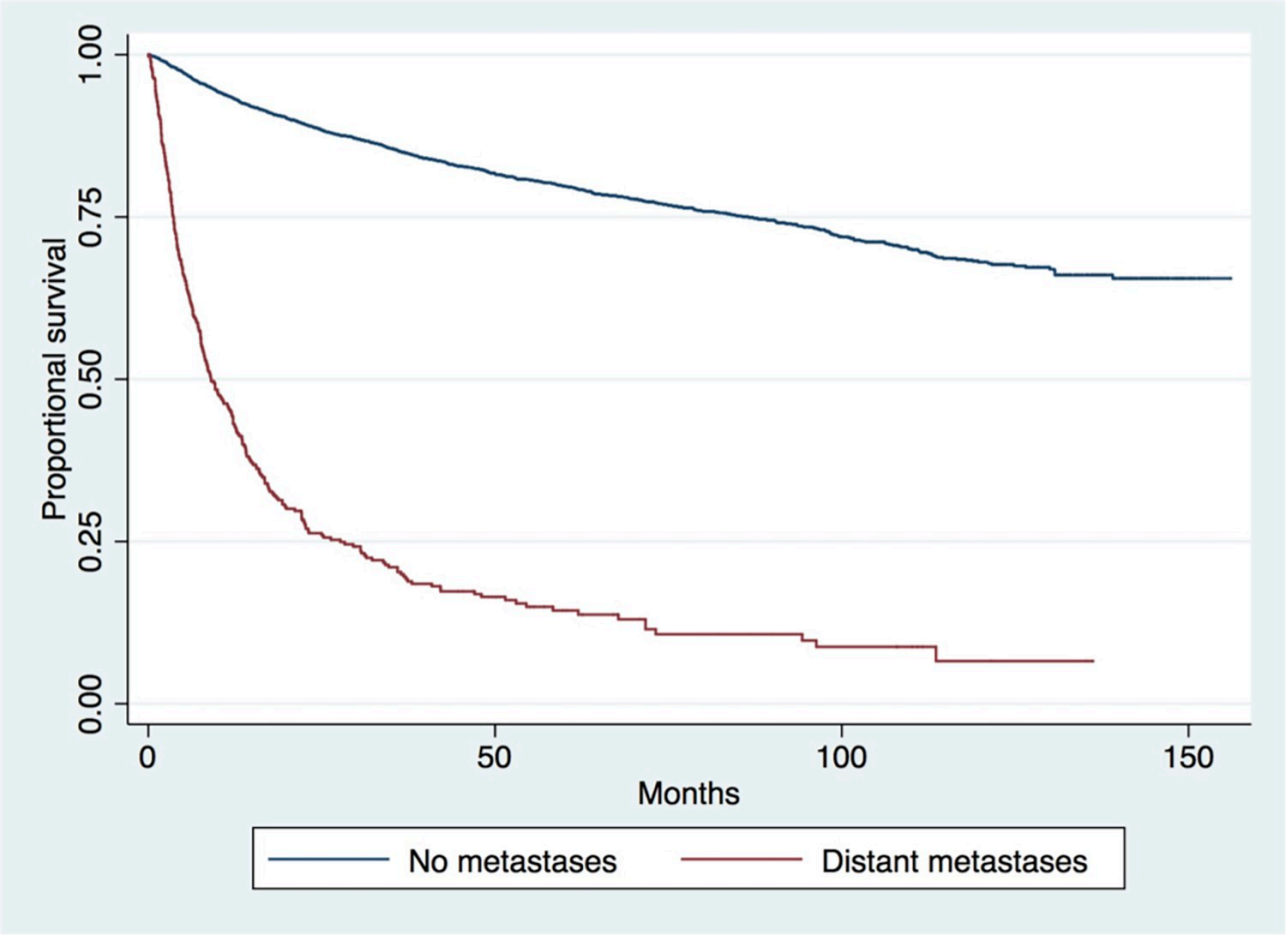
Long-term survival of all patients with and without distant metastases at the time of presentation (p<0.001).

**Fig 3:**
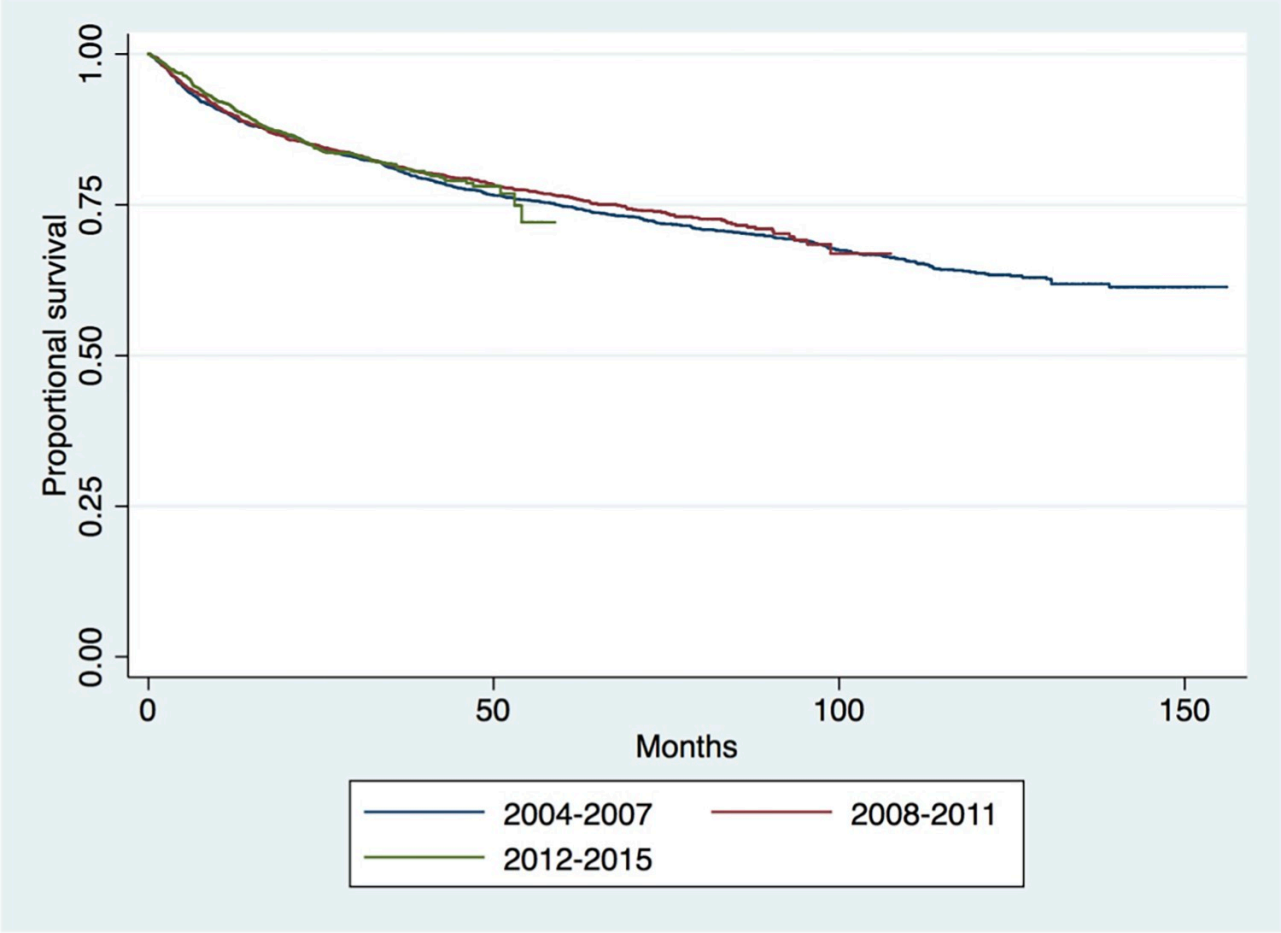
Long-term survival of patients in the three era groups (p = 0.742).

Kaplan-Meier survival analysis showed the worst survival outcomes in the axial cohort and the best outcomes in the “other” group (p<0.001) (Fig 1). Patients with distant metastases at presentation (p<0.001) (Fig 2) had worse survival outcomes. Survival was not significantly increased between older (2004–2007) and more recent years (2012–2016) of treatment (p = 0.742) (Fig 3).

## Discussion

Chondrosarcoma is the second most common primary osseous tumor behind osteosarcoma and makes up about 25% of the total prevalence of bone sarcomas [6]. The gold standard treatment for chondrosarcomas is surgical excision in light of limited responsiveness to radiotherapy and chemotherapy [9]. Prior chondrosarcoma survival studies have been of limited age or from many years ago thus, the currently presented study analyzed 5,329 chondrosarcoma patients from the NCDB database to address the above-noted literature limitation.

Chondrosarcoma has been shown to have the highest 5-year survival rate of the three most common primary sarcomas of the bone [8]. Similar to these prior studies, our study showed an overall 5-year survival rate of 75.6% (prior studies between 72–76%) and 10-year survival rates of 64.18% (prior study 69%) [6, 8, 9]. The current study also found 1-year survival to be as high as 90.38% overall.

While chondrosarcoma maintains a relatively high 5- and 10-year survival rates compared to other sarcomas, both 5 and 10-year survival rates have made minimal progress over the study period with no significant difference between the three eras studied (2004–2007, 2008–2011, and 2012–2015). Further, with the exception of axial cohort 2012–2015 data, the current data shows no significant improvement from 2004 to 2015. However, although the early trajectory seen in Fig 3 shows patients in the later era group to be similar to prior era groups, 5- or 10-year data and the data are incomplete based on years available for follow-up. All this considered, the lack of demonstrated improvement in chondrosarcoma survival rates, supports the continued reliance on traditional treatment algorithms.

Appendicular location was found to be associated with a better prognosis relative to axial location. Giuffrida et al., using the SEER program database from 1973–2003 and other more recent studies have reported results consistent with this finding [9, 16, 21]. As the primary goal of surgical treatment is negative margins, the anatomic limitations of pelvis / spine lesions makes this objective more challenging to achieve [22].

Metastasis at time of diagnosis seems to represent an additional poor predictor for long term survival according to our data. Previous studies have also found the relative 5-year survival to drop precipitously from 75.2% to 28.4% with the presence of metastasis [8–10]. This marked decline in survival rate is suggestive of a more aggressive phenotype or wider spread disease in the setting of metastasis that may be influential in the course of the disease [9, 10].

In the metastatic setting, while surgical intervention remains the most established means of intervention, the implementation of radiotherapy was indicative of improved outcomes. Corroborating research supports the suggestion of possible benefit from radiation, chemotherapy in the setting of metastatic chondrosarcoma [6]. Despite the decreased likelihood of death in our axial cohort with surgical and radiotherapy intervention, many studies remain doubtful of the benefit reported by radiotherapy, identifying only benefit when compared to supportive care [6, 23, 24] or not at all [25]. The role of radiation and chemotherapy in these settings requires further study.

Studies of the treatment algorithms for chondrosarcoma have reached a plateau, with no improvement in survival rates of patients with chondrosarcoma over the last 30 years [16], further corroborated by the current study’s findings. Despite this plateau, surgical treatment remains the most effective and common intervention requiring the surgical excision of the tumor. The significance of tumor location and metastasis at presentation prompt a focus on the importance of improved surgical technique as a potential means to improving outcomes. Associated with worse outcomes due to difficult surgical sites and more widespread trajectory, computer assisted surgery (CAS) may offer assistance in ensuring adequate margins in difficult tumor resections and ensure avoidance of inadvertent tumor perforation [26, 27].

When interpreting the results of this investigation, there are several limitations that need to be taken into account. Just like any large database study, there is always a possibility of mis-collection of data, missing data, or bias of data collection. Due to NCDB data use agreement, the analysis of small sample size sub-cohorts could not be performed so certain sub-categories were not examined. For example, the majority of patients (64%) had no residual tumor and margin data was unavailable for more than 26% of individuals thus limiting their analysis. Additionally, the study is inherently limited in follow-up for the group treated in the later set of years studied. Finally, details of the surgical, radiation, and chemotherapy protocols were not available.

## Conclusions

In conclusion, this review of chondrosarcoma patients from 2004 to 2015 captured over five thousand patients from the NCDB, demonstrating the continued role of surgical intervention as the mainstay of treatment for both appendicular and axial chondrosarcomas. After thirty years, and the continued development of novel therapeutic approaches, we demonstrated that the long-term survival for chondrosarcomas has remained insignificant and continues to rely on surgical treatment. Poor prognostic factors such as distant metastasis and axial involvement that hinder the means of surgical intervention have been shown to decrease long-term survival for patients. These findings suggest the clinical importance of surgery directing our research and attention to the development of novel surgical approaches. Computer assisted surgery offers one possible means to improve the delivery of surgical interventions.

## References

[1] Chow WA. Chondrosarcoma: biology, genetics, and epigenetics. F1000Res. 2018;7. Epub 2018/12/07. doi: 10.12688/f1000research.15953.1 ; PubMed Central PMCID: PMC6248264. 30519452 PMC6248264

[2] van Oosterwijk JG, Anninga JK, Gelderblom H, Cleton-Jansen AM, Bovée JV. Update on targets and novel treatment options for high-grade osteosarcoma and chondrosarcoma. Hematol Oncol Clin North Am. 2013;27(5):1021–48. Epub 2013/10/08. doi: 10.1016/j.hoc.2013.07.012 . 24093174

[3] Hartley AL, Blair V, Harris M, Birch JM, Banerjee SS, Freemont AJ, et al. Sarcomas in north west England: II. Incidence. Br J Cancer. 1991;64(6):1145–50. Epub 1991/12/01. doi: 10.1038/bjc.1991.479 ; PubMed Central PMCID: PMC1977876. 1662534 PMC1977876

[4] Polychronidou G, Karavasilis V, Pollack SM, Huang PH, Lee A, Jones RL. Novel therapeutic approaches in chondrosarcoma. Future Oncol. 2017;13(7):637–48. Epub 2017/01/31. doi: 10.2217/fon-2016-0226 . 28133974

[5] Leddy LR, Holmes RE. Chondrosarcoma of bone. Cancer treatment and research. 2014;162:117–30. Epub 2014/07/30. doi: 10.1007/978-3-319-07323-1_6 . 25070233

[6] Andreou D, Ruppin S, Fehlberg S, Pink D, Werner M, Tunn PU. Survival and prognostic factors in chondrosarcoma: results in 115 patients with long-term follow-up. Acta Orthop. 2011;82(6):749–55. Epub 2011/11/10. doi: 10.3109/17453674.2011.636668 ; PubMed Central PMCID: PMC3247897. 22066552 PMC3247897

[7] Nguyen MT, Jiang YQ, Li XL, Dong J. Risk Factors for Incidence and Prognosis in Chondrosarcoma Patients with Pulmonary Metastasis at Initial Diagnosis. Med Sci Monit. 2019;25:10136–53. Epub 2019/12/31. doi: 10.12659/MSM.919184 ; PubMed Central PMCID: PMC6951109. 31885034 PMC6951109

[8] Damron TA, Ward WG, Stewart A. Osteosarcoma, chondrosarcoma, and Ewing’s sarcoma: National Cancer Data Base Report. Clinical orthopaedics and related research. 2007;459:40–7. Epub 2007/04/07. doi: 10.1097/BLO.0b013e318059b8c9 . 17414166

[9] Wang Z, Chen G, Chen X, Huang X, Liu M, Pan W, et al. Predictors of the survival of patients with chondrosarcoma of bone and metastatic disease at diagnosis. J Cancer. 2019;10(11):2457–63. doi: 10.7150/jca.30388 . 31258751 PMC6584356

[10] Leerapun T, Hugate RR, Inwards CY, Scully SP, Sim FH. Surgical management of conventional grade I chondrosarcoma of long bones. Clin Orthop Relat Res. 2007;463:166–72. Epub 2007/07/17. doi: 10.1097/BLO.0b013e318146830f . 17632422

[11] Lee FY, Mankin HJ, Fondren G, Gebhardt MC, Springfield DS, Rosenberg AE, et al. Chondrosarcoma of bone: an assessment of outcome. The Journal of bone and joint surgery American volume. 1999;81(3):326–38. Epub 1999/04/13. doi: 10.2106/00004623-199903000-00004 . 10199270

[12] Gelderblom H, Hogendoorn PC, Dijkstra SD, van Rijswijk CS, Krol AD, Taminiau AH, et al. The clinical approach towards chondrosarcoma. Oncologist. 2008;13(3):320–9. Epub 2008/04/02. doi: 10.1634/theoncologist.2007-0237 . 18378543

[13] Evans HL, Ayala AG, Romsdahl MM. Prognostic factors in chondrosarcoma of bone: a clinicopathologic analysis with emphasis on histologic grading. Cancer. 1977;40(2):818–31. Epub 1977/08/01. doi: 10.1002/1097-0142(197708)40:2&lt;818::aid-cncr2820400234&gt;3.0.co;2-b . 890662

[14] Amer KM, Munn M, Congiusta D, Abraham JA, Basu Mallick A. Survival and Prognosis of Chondrosarcoma Subtypes: SEER Database Analysis. J Orthop Res. 2020;38(2):311–9. doi: 10.1002/jor.24463 PubMed Central PMCID: PMC31498474. 31498474

[15] Gao Z, Lu T, Song H, Gao Z, Ren F, Ouyang P, et al. Prognostic factors and treatment options for patients with high-grade chondrosarcoma. Med Sci Monit. 2019;25:8952–67. doi: 10.12659/MSM.917959 PubMed Central PMCID: PMC31765367. 31765367 PMC6894367

[16] Giuffrida AY, Burgueno JE, Koniaris LG, Gutierrez JC, Duncan R, Scully SP. Chondrosarcoma in the United States (1973 to 2003): an analysis of 2890 cases from the SEER database. J Bone Joint Surg Am. 2009;91(5):1063–72. Epub 2009/05/05. doi: 10.2106/JBJS.H.00416 . 19411454

[17] Bilimoria KY, Stewart AK, Winchester DP, Ko CY. The National Cancer Data Base: a powerful initiative to improve cancer care in the United States. Ann Surg Oncol. 2008;15(3):683–90. Epub 2008/01/10. doi: 10.1245/s10434-007-9747-3 ; PubMed Central PMCID: PMC2234447. 18183467 PMC2234447

[18] Merkow RP, Rademaker AW, Bilimoria KY. Practical Guide to Surgical Data Sets: National Cancer Database (NCDB). JAMA surgery. 2018;153(9):850–1. Epub 2018/04/05. doi: 10.1001/jamasurg.2018.0492 . 29617542

[19] Surgeons ACo. About the National Cancer Database 2019 [December 23, 2019]. Available from: https://www.facs.org/quality-programs/cancer/ncdb/about.

[20] Sedgwick P. Incidence rate ratio. BMJ. 2010;341:c4804. doi: 10.1136/bmj.c4804 20810482

[21] Bindiganavile S, Han I, Yun JY, Kim H-S. Long-term Outcome of Chondrosarcoma: A Single Institutional Experience. Cancer Res Treat. 2015;47(4):897–903. Epub 2015/02/12. doi: 10.4143/crt.2014.135 . 25687868 PMC4614192

[22] Catanzano AA, Kerr DL, Lazarides AL, Dial BL, Lane WO, Blazer DG, et al. Revisiting the Role of Radiation Therapy in Chondrosarcoma: A National Cancer Database Study. Sarcoma. 2019;2019:4878512. doi: 10.1155/2019/4878512 31736653 PMC6815626

[23] van Maldegem AM, Bovée JV, Gelderblom H. Comprehensive analysis of published studies involving systemic treatment for chondrosarcoma of bone between 2000 and 2013. Clin Sarcoma Res. 2014;4:11-. doi: 10.1186/2045-3329-4-11 . 25126409 PMC4131227

[24] Arshi A, Sharim J, Park DY, Park HY, Bernthal NM, Yazdanshenas H, et al. Chondrosarcoma of the Osseous Spine: An Analysis of Epidemiology, Patient Outcomes, and Prognostic Factors Using the SEER Registry From 1973 to 2012. Spine. 2017;42(9):644–52. doi: 10.1097/BRS.0000000000001870 . 28441682 PMC5561726

[25] Lewis JJ, Leung D, Woodruff JM, Brennan MF. Retroperitoneal soft-tissue sarcoma: analysis of 500 patients treated and followed at a single institution. Annals of surgery. 1998;228(3):355–65. doi: 10.1097/00000658-199809000-00008 . 9742918 PMC1191491

[26] Abe K, Yamamoto N, Hayashi K, Takeuchi A, Miwa S, Igarashi K, et al. The usefulness of wide excision assisted by a computer navigation system and reconstruction using a frozen bone autograft for malignant acetabular bone tumors: a report of two cases. BMC Cancer. 2018;18(1):1036. doi: 10.1186/s12885-018-4971-8 30355277 PMC6201638

[27] Gerbers JG, Stevens M, Ploegmakers JJ, Bulstra SK, Jutte PC. Computer-assisted surgery in orthopedic oncology. Acta Orthop. 2014;85(6):663–9. Epub 2014/08/20. doi: 10.3109/17453674.2014.950800 . 25140984 PMC4259032

